# A new large oregoniid spider crab of the genus *Pleistacantha* Miers, 1879, from the Bay of Bengal, India (Crustacea, Brachyura, Majoidea)

**DOI:** 10.3897/zookeys.716.21349

**Published:** 2017-11-29

**Authors:** Peter K. L. Ng, Raveendhiran Ravinesh, S. Ravichandran

**Affiliations:** 1 Lee Kong Chian Natural History Museum, 2 Conservatory Drive, National University of Singapore, Singapore 117377, Republic of Singapore; 2 Department of Aquatic Biology & Fisheries, University of Kerala, Kariavattom, Thiruvananthapuram – 695581, Kerala, India; 3 Center of Advanced Study in Marine Biology, Annamalai University, Parangipettai 608 502, India

**Keywords:** deep-water, Indian Ocean, new species, Oregoniidae, *Pleistacantha*, taxonomy

## Abstract

A new species of deep-water oregoniid spider crab of the genus *Pleistacantha* Miers, 1879, is described from the Indian Ocean. The species is distinct in possessing a prominently inflated carapace in which the median parts of the branchial regions almost meet. It can also be distinguished from its closest congeners, *P. moseleyi* (Miers, 1885), *P. pungens* (Wood-Mason, in Wood-Mason & Alcock, 1891), and *P. ori* Ahyong & Ng, 2007, in its more elongate and less spinose chelipeds and ambulatory legs, shorter third maxilliped, trapezoidal male pleon and a male first gonopod which is relatively stout with a short subdistal dorsal papilla.

## Introduction

The majoid genus *Pleistacantha* Miers, 1879, currently contains 12 species (Ahyong and Lee 2006; Ahyong and Ng 2007; Ng et al. 2008). While many earlier authors treat *Pleisticanthoides* Yokoya, 1933, and *Parapleisticantha* Yokoya, 1933, as junior synonyms of *Pleistacantha* Miers, 1879 (Ng et al. 2008), these genera are now regarded as distinct genera (see Ng and Richer de Forges 2012; Richer de Forges et al. 2013, respectively). Although *Pleistacantha* has been traditionally classified with the Inachidae MacLeay, 1838 (see Ng et al. 2008), Marco-Herrero et al. (2013) used molecular, larval and morphological evidence to argue that this genus as well as *Bothromaia* Williams & Moffitt, 1991, *Ergasticus* A. Milne-Edwards, 1882, *Parapleisticantha* Yokoya, 1933, and *Pleisticanthoides* Yokoya, 1933, should be transferred to the Oregoniidae Garth, 1958, instead, and in its own subfamily, the Pleistacanthinae Števčić, 2005. This classification is followed here.

The ports of south India with provisions for landing the bycatch of deep sea trawlers are proving to be a major source of rare systematic material for brachyuran studies, and several interesting taxa have been recorded in recent years (e.g., Ng and Kumar 2015, 2016; Mendoza and Suvarna Devi 2017; Ng et al. 2017; Prema et al. 2017). Among the material studied recently is a new species of *Pleistacantha* which is described here. While superficially resembling *P. moseleyi* (Miers, 1885), *P. pungens* (Wood-Mason, in Wood-Mason and Alcock 1891), and *P. ori* Ahyong & Ng, 2007, it has a markedly more inflated carapace, more elongate and less spinose chelipeds and ambulatory legs, a short third maxilliped and trapezoidal male pleonal shape as well as a diagnostic male first gonopod which is relatively stout with a short subdistal dorsal papilla.

## Materials and methods

Specimens examined are deposited in the University of Kerala (**DABFUK**), India; Centre of Advanced study in Marine Biology, Annamalai University (**CASAU**), Parangipettai, Tamil Nadu, India; and the Zoological Reference Collection (**ZRC**) of the Lee Kong Chian Natural History Museum, National University of Singapore. The morphological terms used mostly follow Ahyong et al. (2005) with changes suggested by Davie et al. (2015).

The following abbreviations are used:

**cl** maximum carapace length (including rostrum);

**cw** carapace width (including spines);

**G1** male first pleopod;

**G2** male second pleopod;

**pcl** pre-rostral carapace length (maximum carapace length excluding rostrum).

All measurements are in millimetres. The ambulatory legs (pereopods 2–5) are abbreviated **P2–5**, respectively.

## Systematics

### Family Oregoniidae Garth, 1958

#### Genus Pleistacantha

Taxon classificationAnimaliaDecapodaMajoidea

Miers, 1879

##### Type species.

*Pleistacantha sanctijohannis* Miers, 1879, by original designation.

#### Pleistacantha kannu sp. n.

Taxon classificationAnimaliaDecapodaMajoidea

http://zoobank.org/9FB362C1-38CF-4532-A1AE-ABCD3A2C5738

Figs 1 , 2 , 4C, D , 5C, D , 6E, F , 7G–I , 8E, F , 9E, F , 10K–N , 11

?Pleistacantha adenicusKazmi 1997: 82, figs 1, 2 (*nomen nudum*).

##### Material examined.

Holotype: male (cl 106.2 mm, pcl 87.4 mm, cw 87.0 mm) (CASAU), Pazhayar, coll. S. Ravichandran et al., 2017. Paratypes: 1 female (cl 83.9 mm, pcl 78.7 mm, cw 72.9 mm) (rostrum broken) (CASAU), 1 ovigerous female (cl 84.4 mm, pcl 69.8 mm, cw 71.5 mm) (all ambulatory legs broken off), 1 female (cl 79.5mm, pcl 65.21 mm, cw 66.9 mm) (each side two pairs of ambulatory legs broken) (CASAU), same data as holotype; 1 ovigerous female (cl 91.3 mm, pcl 81.2 mm, cw 75.5 mm), 2 females (cl 85.2 mm, pcl 75.0 mm, cw 70.4 mm; cl 87.3 mm, pcl 77.2 mm, cw 72.5 mm) (DABFUK), Tuticorin fishing port, India, coll. R. Ravinesh, March 2017; 1 female (cl 90.4 mm, pcl 80.2 mm, cw 73.9 mm) (DABFUK), Muttam Fishing Harbour, coll. A. B. Kumar, 14 October 2015. All localities from state of Tamil Nadu, India.

**Figure 1.**
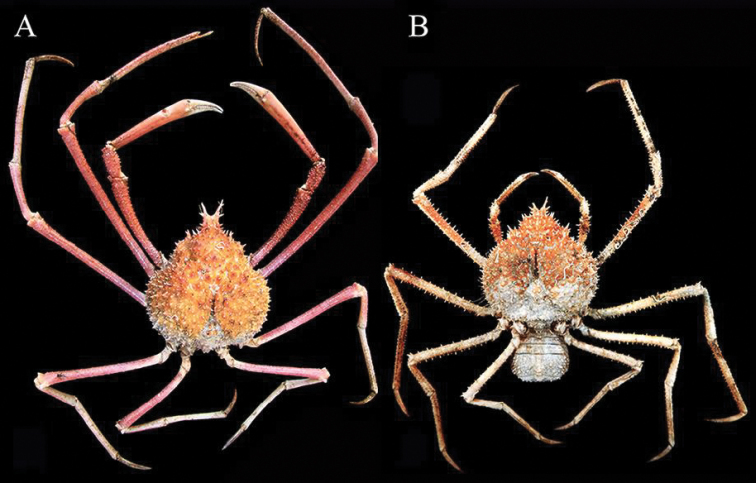
*Pleistacantha kannu* sp. n., colour in life. **A** holotype male (cl 106.2 mm, cw 87.0 mm) (CASAU), India **B** paratype ovigerous female (cl 84.4 mm, cw 71.5 mm) (CASAU), India.

##### Comparative material.

*Pleistacantha moseleyi* (Miers, 1885): 1 male (cl 82.5 mm, pcl 61.6 mm, cw 61.6 mm) (ZRC 2005.117), Maribohoc Bay, Panglao, Bohol. Philippines, 100–300 m, coll. T. J. Arbasto, November 2003–March 2004. *Pleistacantha pungens* (Wood-Mason, in Wood-Mason and Alcock 1891): 1 male (cl 94.0 mm, pcl 66.9 mm, cw 67.6 mm) (ZRC 2016.23), Ayeyarwady Delta, station 58, 14.3225°N, 93.7405°E, off Myanmar, Andaman Sea, 265–268.5 m, bottom trawl, coll. EAF-NANSEN Project (Myanmar Cruise), 9 May 2015; 1 female (cl 45.2 mm, pcl 30.8 mm, cw 29.8 mm) (ZRC 2016.24), Ayeyarwady Delta, station 68, 14.06216667°N, 94.31816667°E, off Myanmar, Andaman Sea, 455–457 m, bottom trawl, coll. EAF-NANSEN Project (Myanmar Cruise), 10 May 2015. *Pleistacantha ori* Ahyong & Ng, 2007: holotype male (cl 146.0 mm, pcl 129.1 mm, cw 106.3 mm) (ZRC 2006.158), off Durban, South Africa, coll. Oceanographic Research Institute, Durban, October 2004; paratypes: 1 male (cl 129.0 mm, pcl 115.0 mm, cw 92.7 mm), 1 ovigerous female (cl 120+ mm, pcl 106.6 mm, cw 83.9 mm), 1 spent female (cl 119.6 mm, pcl 104.9 mm, cw 84.5 mm) (ZRC 2006.0159), same data as holotype.

##### Etymology.

Name after the late Professor T. Kannupandi, an influential crustacean worker from the Centre of Advanced Study in Marine Biology in Annamalai University. The name, a shortened version of his family name, is used as a noun in apposition.

##### Diagnosis.

**Figure 2.**
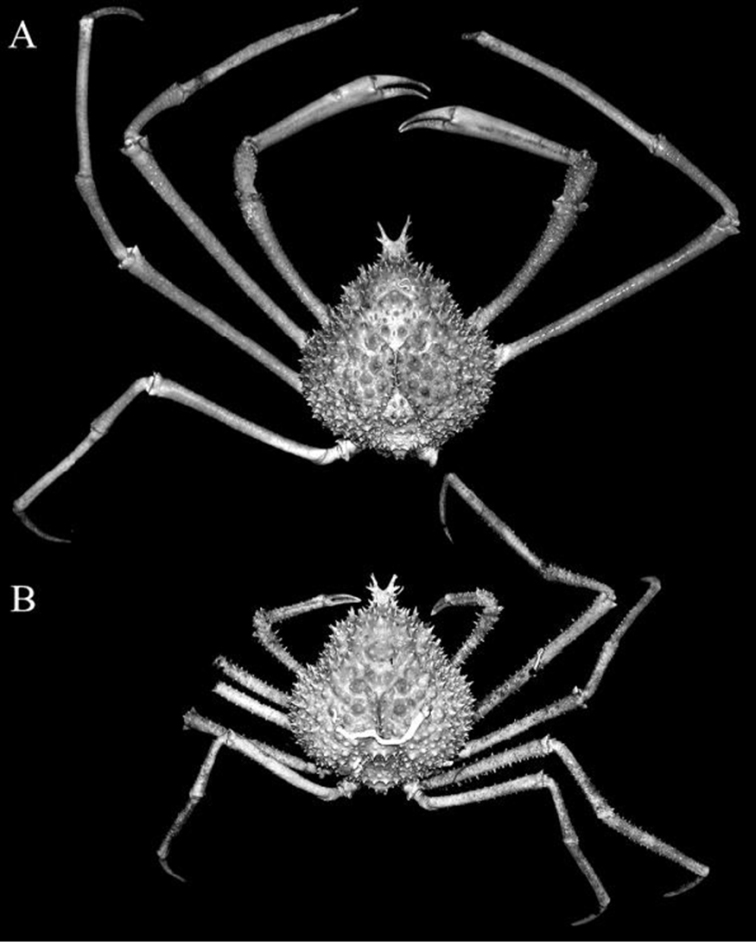
*Pleistacantha kannu* sp. n. **A** holotype male (cl 106.2 mm, cw 87.0 mm) (CASAU), India **B** paratype ovigerous female (cl 91.3 mm, cw 75.5 mm) (DABFUK), India.

Carapace broadly pyriform, postrostral carapace length equal to or slightly longer than carapace width (ratio 1.0–1.1) (Figs 2A, B, 4C, D); dorsal carapace surface with short spines with relatively wider bases (Figs 2A, B, 4C, D, 5C, D); gastric regions strongly swollen (Figs 2A, B, 4C, D, 5C, D); branchial regions strongly swollen laterally and dorsally; medially separated by narrow space, area without spines, spines on margins of regions overlapping (Figs 2A, B, 4C, D, 5C, D); posterior carapace margin convex (Fig. 4C); rostrum relatively short; gently divergent, directly obliquely laterally, not curving upwards (Figs 2A, B, 4C, D, 5C, D, 6E); interantennular spine short, tip bifurcated with shallow concavity between short processes (Figs 6E, F); lateral margins of posterior margin of epistome strongly concave (Fig. 7G, H); ischium of third maxilliped short (Fig. 7I); adult male cheliped elongate, merus and chela slender (Figs 2A, 8F); surface of adult male chela mostly smooth, proximal part with short tubercles or granules, without long spines (Figs 2A, 8E, F); male anterior thoracic sternum relatively broad; surface with numerous blunt and sharp tubercles, never spines (Fig. 9E); male pleon transversely wide; distinctly trapezoidal; surface with numerous blunt and sharp tubercles, never spines (Fig. 9F); G1 relatively stout; distal part gently curved; subdistal dorsal papilla short (Fig. 10K–M).

##### Description of holotype male.

**Figure 3.**
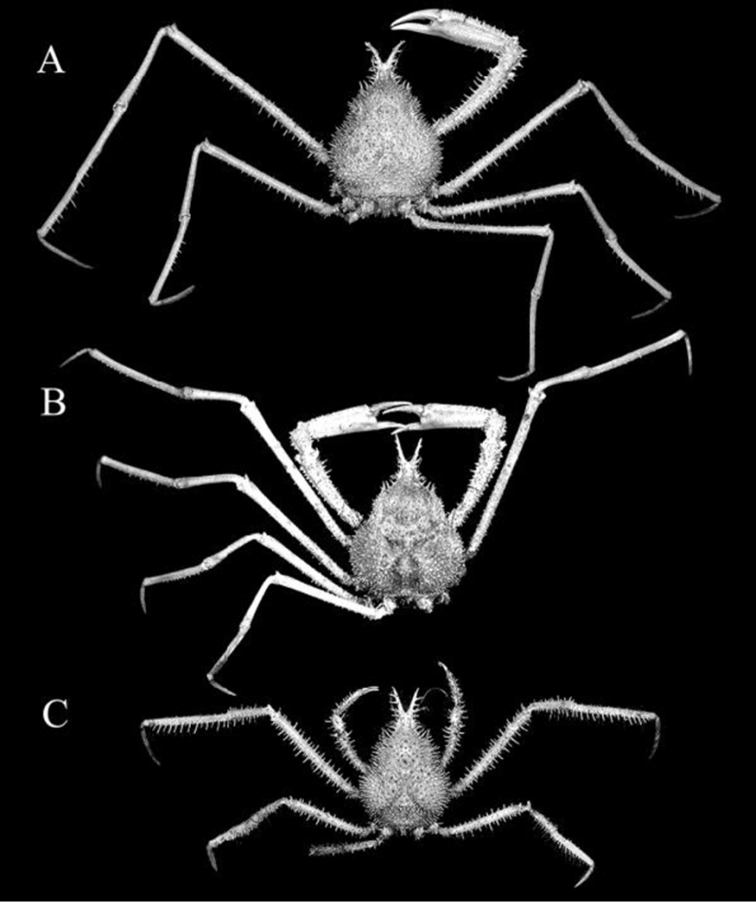
**A** *Pleistacantha moseleyi* (Miers, 1885), male (cl 82.5 mm, cw 61.6 mm) (ZRC 2005.117), Philippines **B** *Pleistacantha pungens* (Wood-Mason, in Wood-Mason and Alcock 1891), male (cl 94.0 mm, cw 67.6 mm) (ZRC 2016.23), Myanmar **C** *Pleistacantha pungens* (Wood-Mason, in Wood-Mason and Alcock 1891), female (cl 45.2 mm, cw 29.8 mm) (ZRC 2016.24), Myanmar.

**Figure 4.**
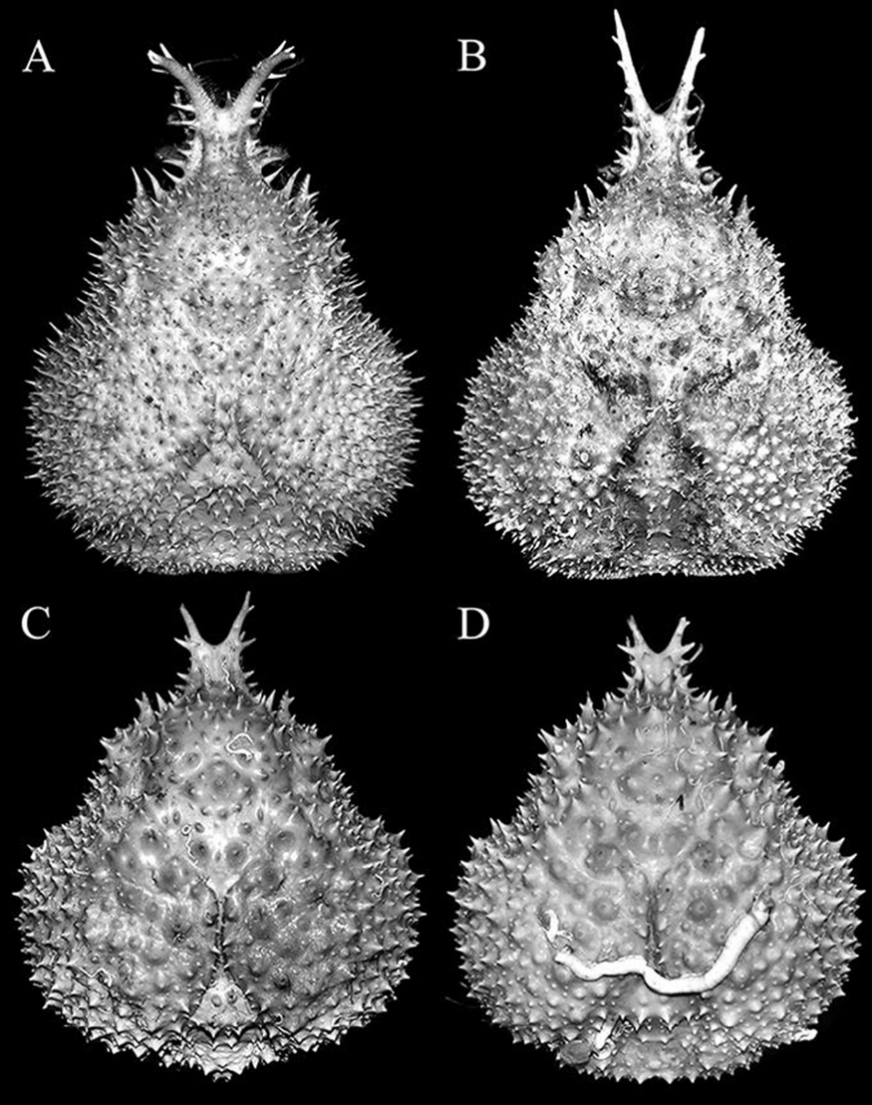
**A** *Pleistacantha moseleyi* (Miers, 1885), male (cl 82.5 mm, cw 61.6 mm) (ZRC 2005.117), Philippines **B** *Pleistacantha pungens* (Wood-Mason, in Wood-Mason and Alcock 1891), male (cl 94.0 mm, cw 67.6 mm) (ZRC 2016.23), Myanmar **C** *Pleistacantha pungens* Wood-Mason, in Wood-Mason and Alcock 1891) **C** *Pleistacantha kannu* sp. n., holotype male (cl 106.2 mm, cw 87.0 mm) (CASAU), India **D** *Pleistacantha kannu* sp. n., paratype ovigerous female (cl 91.3 mm, cw 75.5 mm) (DABFUK), India.

**Figure 5.**
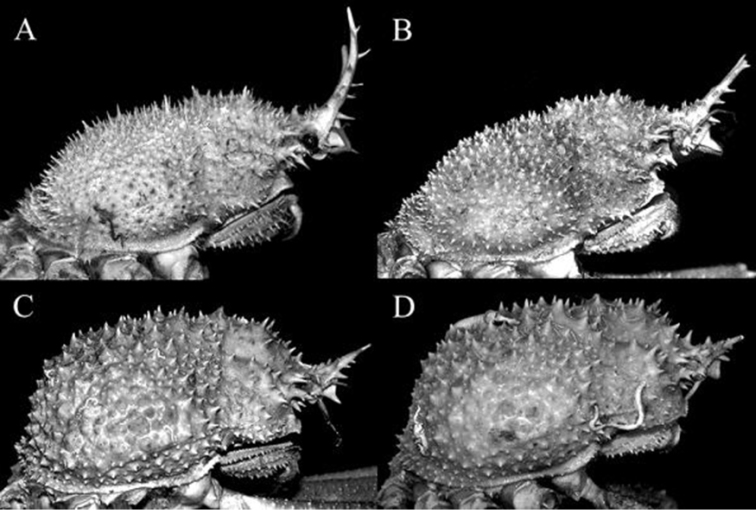
**A** *Pleistacantha moseleyi* (Miers, 1885), male (cl 82.5 mm, cw 61.6 mm) (ZRC 2005.117), Philippines **B** *Pleistacantha pungens* Wood-Mason, in Wood-Mason and Alcock 1891), male (cl 94.0 mm, cw 67.6 mm) (ZRC 2016.23), Myanmar **C** *Pleistacantha kannu* sp. n., holotype male (cl 106.2 mm, cw 87.0 mm) (CASAU), India **D** *Pleistacantha kannu* sp. n., paratype ovigerous female (cl 91.3 mm, cw 75.5 mm) (DABFUK), India.

**Figure 6.**
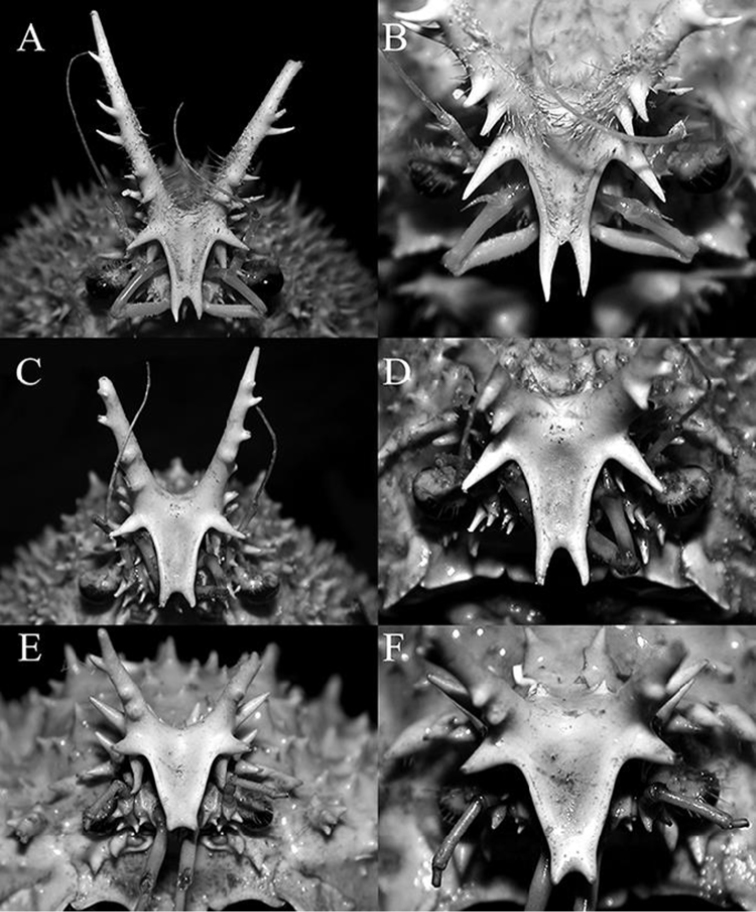
**A, B** *Pleistacantha moseleyi* (Miers, 1885), male (cl 82.5 mm, cw 61.6 mm) (ZRC 2005.117), Philippines **C, D** *Pleistacantha pungens* Wood-Mason, in Wood-Mason and Alcock 1891), male (cl 94.0 mm, cw 67.6 mm) (ZRC 2016.23), Myanmar **E, F** *Pleistacantha kannu* sp. n., holotype male (cl 106.2 mm, cw 87.0 mm) (CASAU), India.

**Figure 7.**
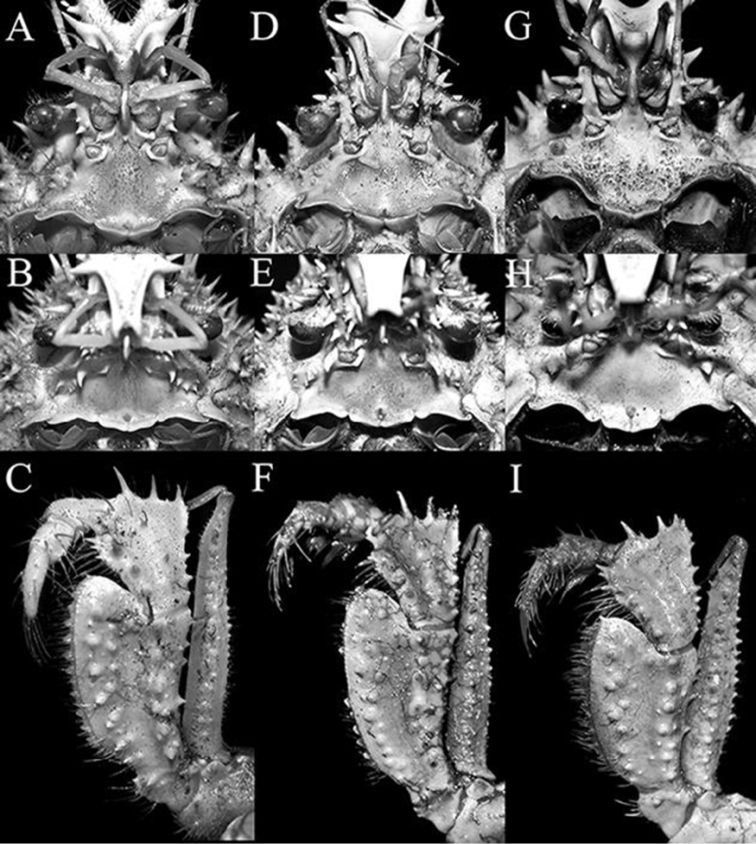
**A–C** *Pleistacantha moseleyi* (Miers, 1885), male (cl 82.5 mm, cw 61.6 mm) (ZRC 2005.117), Philippines **D–F** *Pleistacantha pungens* Wood-Mason, in Wood-Mason and Alcock 1891), male (cl 94.0 mm, cw 67.6 mm) (ZRC 2016.23), Myanmar **G–I** *Pleistacantha kannu* sp. n., holotype male (cl 106.2 mm, cw 87.0 mm) (CASAU), India. **A**, **D**, **G** epistome, antennae and antennules; **B, E, H** epistome; **C, F, I** left third maxilliped.

Carapace broadly pyriform, postrostral carapace length almost equal to carapace width (Figs 2A, 4C). Rostral spines short, 0.2 times postrostral carapace length; basal half completely fused, medially gently divergent laterally, not curving upwards; dorsal surface with three small low dorsal spinules; lateral margin with one or two ventral (excluding basal) spines; with three equally spaced lateral spines, median one largest, at junction of diverging spine (Fig. 3C). Interantennular spine strongly bent downwards, surface concave, bifurcated distally, depth of bifurcation shallow, processes short; distal margin of antennular sinus produced to form prominent ventrolaterally directed spine (Figs 6E, F, 7G). Orbital margin with three large spines increasing in size posteriorly, including intercalated and postorbital spines (Fig. 4C). Hepatic spine large, anteriorly directed, with 2–4 small accessory spines (Fig. 4C). Dorsal surface covered with short conical spines with broad bases and acute tubercles (Figs 2A, 4C, 5C). Branchial regions markedly swollen dorsally and laterally, inner margins almost touching along carapace midline, with associated spines overlapping opposite region, regions separated by narrow longitudinal channel; cardiac region not prominently swollen, with two rows of six short spines in total; intestinal region not well demarcated, region appears depressed (Figs 2A, 4C, 5C). Posterior carapace margin convex (Fig. 4C). Sub-branchial region covered with short, stout spinules; pteryogostomial region with scattered sharp tubercles (Fig. 5C).

Proepistome with ventrally directed, laterally flattened tooth, tip rounded; margin lateral to antennal gland aperture (infraorbital margin) with 2 long spines; anterolateral angle of buccal cavity flared, lobiform, margin with two or three low teeth (Fig. 7G, H). Eye short, when folded back into ‘orbit’, not reaching antennal gland aperture; ocular peduncle short, with 2 granules on subdistal surface adjacent to cornea. Basal antennular article with two short spines. Basal antennal article elongate, rectangular, outer margin with two short spines, mesial margin with two short spines; next article elongate, with two sharp tubercles (Fig. 7G). Epistome wide; posterior margin with lateral margins strongly concave; median lobe subtruncate, separated by deep median fissure, separated from lateral margin by V-shaped cleft (Fig. 7G, H).

Third maxilliped merus almost as wide as ischium; meral surface spinose, with long slender spines on either side of carpal articulation, anterolateral angle triangular, produced, with spinose margins; ischium short, with dentate margins, surface with two longitudinal rows of tubercles separated by shallow median sulcus; exopod relatively slender, outer surface and outer margin each with row of short spines (Fig. 7I).

**Figure 8.**
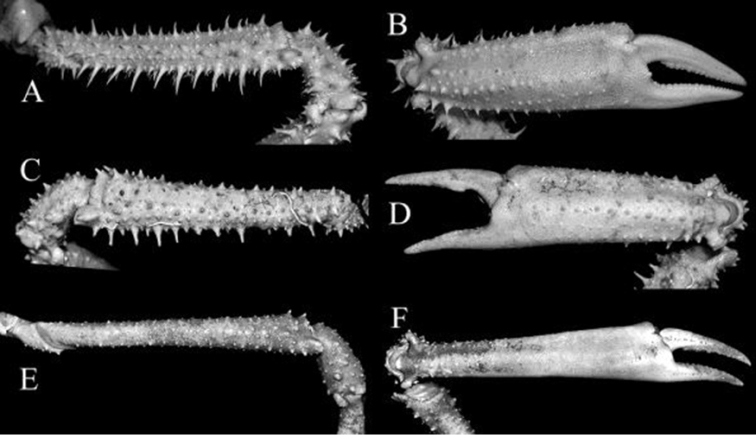
**A, B** *Pleistacantha moseleyi* (Miers, 1885), male (cl 82.5 mm, cw 61.6 mm) (ZRC 2005.117), Philippines **C, D** *Pleistacantha pungens* Wood-Mason, in Wood-Mason and Alcock 1891), male (cl 94.0 mm, cw 67.6 mm) (ZRC 2016.23), Myanmar **E, F** *Pleistacantha kannu* sp. n., holotype male (cl 106.2 mm, cw 87.0 mm) (CASAU), India. **A, C, E** outer view of merus of cheliped; **B, D, F** outer view of chela.

Male cheliped elongate, slender, symmetrical (Fig. 2A). Male chela elongated, stout, not distinctly inflated; distal two-thirds relatively smooth, proximal third with tubercles and low spines; occlusal margins of dactylus and pollex with blunt, obtuse teeth, not forming distinct gape when closed; carpus and merus with numerous sharp tubercles and granules along margins and surfaces; merus elongate, slender with distal half wider than proximal part (Figs 2A, 8E, F). Ambulatory legs (P2–5) long, slender, decreasing in length posteriorly (Fig. 2A). Surfaces of propodus, carpus and merus of P2–4 granular, with short tubercles or granules, not spinose; dactylus covered with dense soft setae and corneous tip (Fig. 2A).

**Figure 9.**
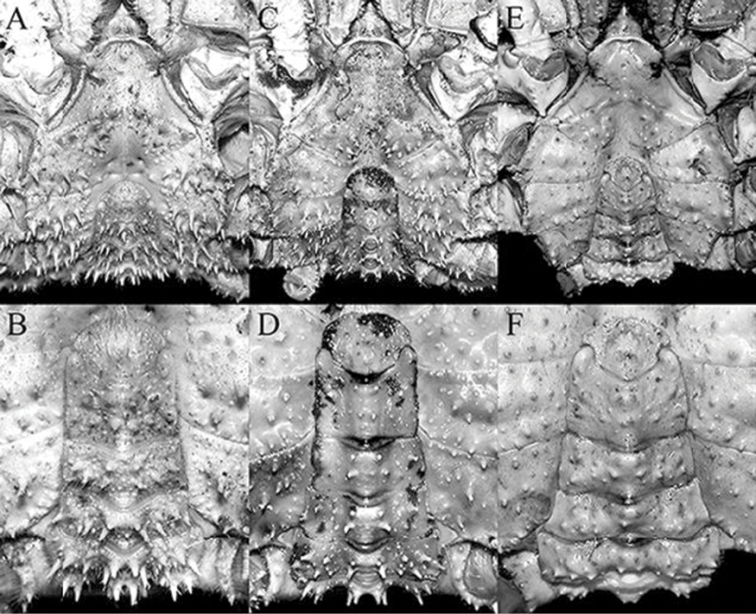
**A, B** *Pleistacantha moseleyi* (Miers, 1885), male (cl 82.5 mm, cw 61.6 mm) (ZRC 2005.117), Philippines **C, D** *Pleistacantha pungens* Wood-Mason, in Wood-Mason and Alcock 1891), male (cl 94.0 mm, cw 67.6 mm) (ZRC 2016.23), Myanmar **E, F** *Pleistacantha kannu* sp. n., holotype male (cl 106.2 mm, cw 87.0 mm) (CASAU), India. **A, C, E** anterior thoracic sternum and pleon; **B, D, F** male pleon.

Anterior thoracic sternum relatively wide transversely (Fig. 9E). Thoracic sternites 1 and 2 fused, forming acutely triangular process; separated from sternite 3 by prominent ridge with concave surface; sternites 3 and 4 fused, anterior part constricted at junction of sternites; sternite 4 with low obliquely transverse ridge lined with tubercles, anterior surface with more prominent tubercles; surfaces of sternites 5–7 with scattered tubercles, some relatively sharp; sternopleonal cavity reaching to suture between sternites 4 and 5 (Fig. 9E, F). Male pleon with six free somites and telson; somites 4–6 trapezoidal, wide; widest at somites 2 and 3; surface tuberculate but not spinose (Fig. 9E, F).

**Figure 10.**
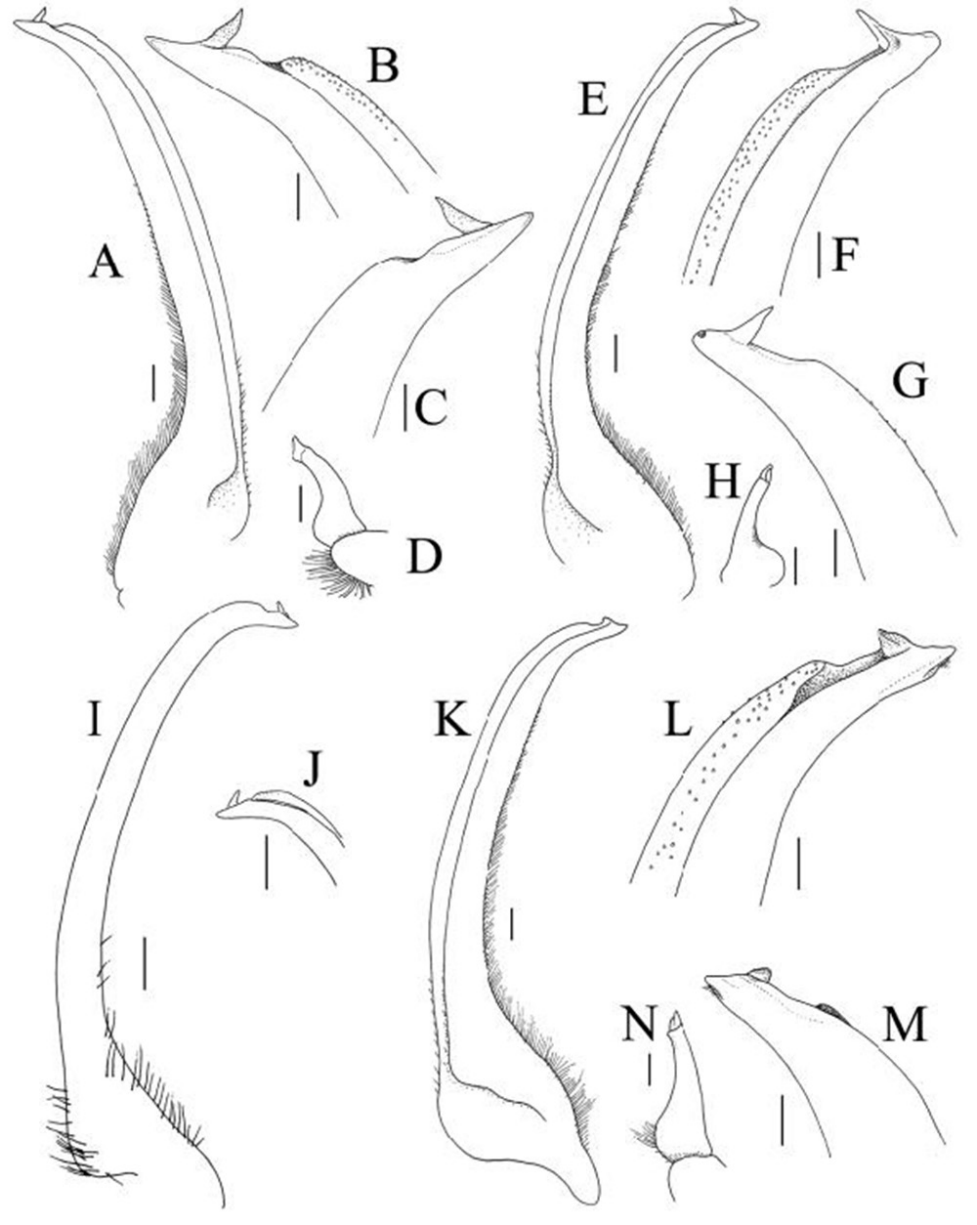
**A–D** *Pleistacantha moseleyi* (Miers, 1885), male (cl 82.5 mm, cw 61.6 mm) (ZRC 2005.117), Philippines **E–H** *Pleistacantha pungens* Wood-Mason, in Wood-Mason and Alcock 1891), male (cl 94.0 mm, cw 67.6 mm) (ZRC 2016.23), Myanmar **I**, **J** *Pleistacantha ori* Ahyong & Ng, 2007, holotype male (cl 146.0 mm, cw 106.3 mm) (ZRC 2006.0158), South Africa (after Ahyong & Ng 2007: fig. 4) **K–N** *Pleistacantha kannu* sp. n., holotype male (cl 106.2 mm, cw 87.0 mm) (CASAU), India. **A–C, I, J** right G1; **D** right G2; **E–G, K–M** left G1; **H, N** left G2. Scales: **A, D, E, H, K, N** 1.0 mm; **B, C, F, G, L, M** 0.5 mm; **I, J** 2.0 mm.

G1 gently curving outwards, relatively shorter, with distal tenth more distinctly curved; subdistal papilla on inner margin short, triangular, shorter than length between papilla base and tip (Fig. 10K–M). G2 short, with basal part dilated; distal part approximately bifurcate, short (Fig. 10N).

##### Females.

**Figure 11.**
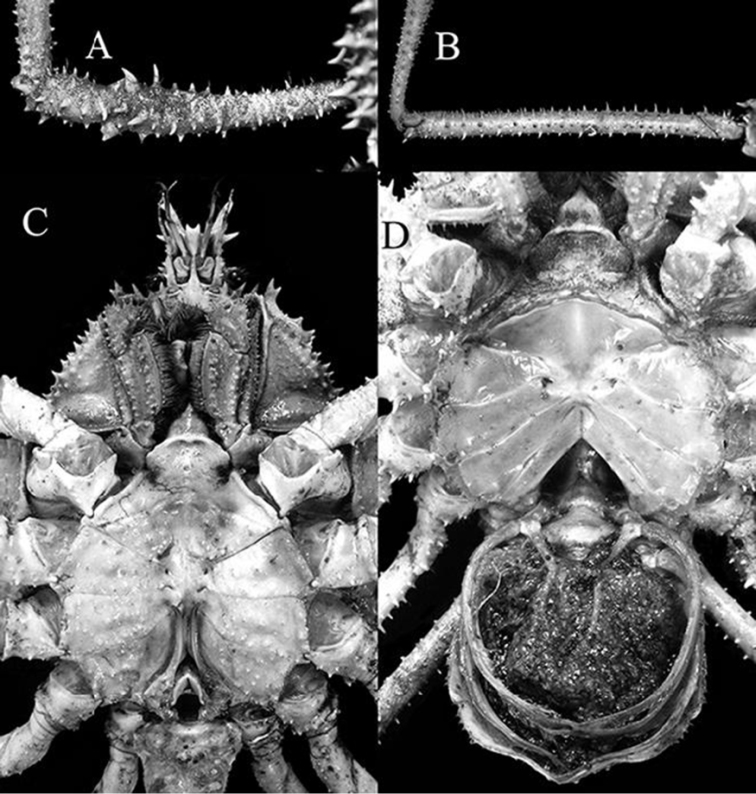
*Pleistacantha kannu* sp. n. **A, B** paratype ovigerous female (cl 91.3 mm, cw 75.5 mm) (DABFUK), India **C** holotype male (cl 106.2 mm, cw 87.0 mm) (CASAU), India **D** paratype ovigerous female (cl 84.4 mm, cw 71.5 mm) (CASAU), India.

The adult females differ from the holotype male in possessing a proportionately shorter rostrum and chelipeds (Figs 1B, 2B, 11A). In addition, the surfaces of the chelipeds and ambulatory legs are covered with more prominent spines and sharper tubercles, with these structures all appearing distinctly spinose (Figs 1B, 2B, 11A, B). The spines and tubercles on the carapace of females (Fig. 4D, 5D) also tend to be relatively more acute compared to those on the male (Fig. 4C, 5C). *Pleistacantha* species are typically sexually dimorphic in these respects (see Grindley 1961; Ahyong & Ng 2007). The female pleon is very broad, and while all the somites and telson appear to be free, they are quite rigid due to their strongly convex shape, forming a dome-like structure (Fig. 11D). The vulvae are large, subovate and positioned on the anterior surface of sternite 6 (Fig. 11D).

##### Colour.

Most of dorsal carapace surface of carapace orange to orange-red (Fig. 1); male chelipeds and ambulatory legs reddish-brown on dorsal surface except for white fingers (Fig. 1A); female chelipeds and ambulatory legs orange and white (Fig. 1B); ventral surfaces dirty white.

##### Remarks.

Ahyong and Ng (2007: 72) recognized a group of three large species of *Pleistacantha* readily distinguished from congeners by possessing a “relatively uniform dorsal carapace spination, in which the dorsal spines are of similar length rather than having several gastric and branchial spines markedly longer than the remainder, in combination with divergent rather than medially appressed rostral spines, and a deeply bifurcate interantennular spine.” *Pleistacantha moseleyi* (Miers, 1885) is known from the Philippines in the western Pacific (Guinot and Richer de Forges 1982, 1986; Ahyong and Lee 2006) while *P. pungens* Wood-Mason, in Wood-Mason and Alcock 1891) and and *P. ori* Ahyong & Ng, 2007, are known from the eastern and western Indian Ocean basins, respectively (Guinot and Richer de Forges 1982, 1986; Ahyong and Ng 2006, 2007). Although most extant literature cite the *Challenger* material as “Miers, 1886”, a number of names like *Pleistacantha* were actually validated by the same author a year earlier (see Froglia and Clark 2011).

The present new species most closely resembles *P. moseleyi* and *P. pungens* in having the branchial regions relatively more swollen, such that the inner margins are close to each other in the midline of the carapace with the gastric and cardiac regions forming an approximate “hour-glass” shape (cf. Ahyong and Ng 2007: 73). In *P. kannu* sp. n., however, the branchial regions are more prominently swollen laterally and dorsally, so much so that the inner margins are now almost adjacent to each other, forming a narrow channel between them (Fig. 4C, D). The margins are so close that the adjacent spines overlap each other (Fig. 4C, D). As the specimens are all comparable in size, the difference in carapace inflation is not size-related. In addition, *P. kannu* can be distinguished from these three congeners in its relatively broader carapace (Figs 2A, B, 4C, D), the spines on the carapace are relatively broader and shorter even in large specimens (Figs 2A, B, 4C, D, 5C, D), the posterior carapace margin is distinctly convex (Fig. 4C), the lateral margins of the posterior margin of the epistome are prominently more concave (Fig. 7G, H), the ischium of the third maxilliped is proportionately shorter (Fig. 7I), the rostral spines are proportionately shorter, gently divergent, not curving upwards (Figs 2A, B, 4C, D, 5C, D, 6E), the interantennular spine is short and while the tip is bifurcated, the processes are short (Figs 6E, F), the male anterior thoracic sternum is proportionately broader (Fig. 9E), the adult male chelipeds are proportionately more slender and longer, with the chela elongate rather than distinctly inflated and mostly smooth (Figs 2A, 8F), the male pleon is broader and more trapezoidal in shape (Fig. 9F), and G1 is not elongate with the distal part not sharply curved and the subdistal dorsal papilla is short (Fig. 10K–M) (Table 1).

**Table 1.** Differences between *Pleistacantha moseleyi* (Miers, 1885), *P. pungens* Wood-Mason, in Wood-Mason and Alcock 1891), *P. ori* Ahyong & Ng, 2007, and *P. kannu* sp. n.

	*Pleistacantha moseleyi*	*Pleistacantha pungens*	*Pleistacantha ori*	*Pleistacantha kannu*
Carapace	Pyriform (Figs 3A, 4A)	Pyriform (Figs 3B, C, 4B)	Pyriform (cf. Ahyong & Ng 2007: fig. 1A, B)	Broadly pyriform (Figs 2A, B, 4C, D)
Spines on dorsal surface of carapace	Acute (Figs 3A, 4A, 5A)	Acute (Figs 3B, C, 4B, 5B)	Relatively acute but short (cf. Ahyong & Ng 2007: fig. 1A, B)	Relatively more obtuse basally (Figs 2A, B, 4C, D, 5C, D)
Gastric regions	Gently swollen (Figs 4A, 5A)	Gently swollen (Figs 4B, 5B)	Gently swollen (cf. Ahyong & Ng 2007: fig. 1A, B)	Strongly swollen (Figs 2A, B, 4C, D, 5C, D)
Branchial regions	Gently swollen laterally and dorsally; medially separated by distinct space and several large short, vertical spines; spines on margins of regions not overlapping (Figs 4A, 5A)	Gently swollen laterally and dorsally; medially separated by distinct space, area without spines; spines on margins of regions not overlapping (Figs 4B, 5B)	Gently swollen laterally and dorsally; medially separated by wide space, area with short spines; spines on margins of regions not overlapping (cf. Ahyong & Ng 2007: fig. 1A, B)	Strongly swollen laterally and dorsally; medially separated by narrow space, area without spines; spines on margins of regions overlapping (Figs 2A, B, 4C, D, 5C, D)
Posterior carapace margin	Gently concave (Fig. 4A)	Gently concave (Fig. 4B)	Distinctly convex (cf. Ahyong & Ng 2007: fig. 1B)	Distinctly convex (Fig. 4C, D).
Rostral spines	Relatively long; strongly divergent; distinctly curving upwards (Figs 3A, 4A, 5A, 6A)	Relatively long; gently divergent; directed obliquely laterally, not curving upwards (Figs 3B, C, 4B, 5B, 6C)	Relatively long; gently divergent; directed obliquely laterally, not curving upwards (cf. Ahyong & Ng 2007: figs 1C, 2B)	Relatively short; gently divergent; directly obliquely laterally, not curving upwards (Figs 2A, B, 4C, D, 5C, D, 6E)
Interantennuar spine	Long, slender; tip deeply bifurcated forming 2 long processes (Figs 6A, B)	Long; tip deeply bifurcated forming 2 long processes (Figs 6C, D)	Long; tip deeply bifurcated forming 2 long processes (cf. Ahyong & Ng 2007: fig. 2D)	Short; tip bifurcated with shallow concavity between short processes (Figs 6E, F)
Posterior margin of epistome	Lateral margins gently concave (Fig. 7A, B)	Lateral margins gently concave (Fig. 7D, E)	Lateral margins gently concave (cf. Ahyong & Ng 2007: fig. 2A)	Lateral margins strongly concave (Fig. 7G, H)
Third maxilliped	Ischium elongate (Fig. 7C)	Ischium elongate (Fig. 7F)	Ischium elongate (cf. Ahyong & Ng 2007: fig. 3C)	Ischium short (Fig. 7I)
Outer surfaces of male chelipeds	With numerous long sharp spines (Figs 3A, 8A, B)	With numerous long sharp spines (Figs 3B, 8E, F)	Mostly smooth, proximal part with short tubercles or granules, without long spines (cf. Ahyong & Ng 2007: fig. 1E)	Mostly smooth, proximal part with short tubercles or granules, without long spines (Figs 2A, 8E, F)
Male merus	Relatively short, stout (Figs 3A, 8A)	Relatively short, stout (Figs 3B, 8C)	Relatively short, stout (cf. Ahyong & Ng 2007: 1A)	Elongate, slender (Figs 2A, 8E)
Male chela	Relatively short, stout (Figs 3A, 8B)	Relatively short, stout (Figs 3B, 8D)	Relatively short, stout (cf. Ahyong & Ng 2007: fig. 1E)	Elongate, slender (Figs 2A, 8F)
Male anterior thoracic sternum	Relatively narrow; surface with numerous sharp posteriorly directed spines (Fig. 9A)	Relatively narrow; surface with sharp posteriorly directed spines and tubercles (Fig. 9C)	Relatively broad; surface with numerous sharp tubercles and short spines (cf. Ahyong & Ng 2007: 2C)	Relatively broad; surface with numerous blunt and sharp tubercles, never spines (Fig. 9E)
Male pleon (somites 4–6)	Relatively narrow transversely; almost rectangular in shape; surface with numerous sharp posteriorly directed spines (Fig. 9B)	Relatively narrow transversely; almost rectangular in shape; surface with sharp posteriorly directed spines and tubercles (Fig. 9D)	Relatively narrow transversely; almost rectangular in shape; surface with sharp posteriorly directed spines and tubercles (cf. Ahyong & Ng 2007: fig. 2C)	Transversely wide; distinctly trapezoidal; surface with numerous blunt and sharp tubercles, never spines (Fig. 9F)
G1	Relatively stout; distal part gently curved; subdistal dorsal papilla long (Fig. 10A–C)	Relatively slender; distal part gently curved; subdistal dorsal papilla long (Fig. 10E–G)	Relatively long, slender; distal part sharply curved; subdistal dorsal papilla long (Fig. 10I, J; Ahyong & Ng 2007: fig. 4)	Relatively stout; distal part gently curved; subdistal dorsal papilla short (Fig. 10K–M)

*Pleistacantha kannu* may be conspecific with a taxon discussed in Kazmi (1997) and identified as “*Pleistacantha adenicus*”. In a review of Pakistani spider crabs, Kazmi (1997) discussed the identity of a berried female of *Pleistacantha* measuring 68 mm in carapace length collected from the mouth of the Gulf of Aden in her collection. In her abstract, she noted that “The occurrence of the genus *Pleistacantha* in the Arabian Sea and its adjacent gulfs is discussed in detail due to presence of a unique female which was taken by *Fridtjof Nansen* Cruise in 1977. This seems to be an undescribed species. At the moment the female is just given as *Pleistacantha* sp1 till more specimens obtained determine its position.” (Kazmi 1997: 79). Later in the paper, the heading for her discussion was written as “DESCRIPTION OF UNDESCRIBED *PLEISTACANTHA*. *Pleistacantha* sp. 1” and she commented that “My specimen is still unnamed and is given here as *P.* sp. 1” (Kazmi 1997: 82). She described and compared the specimen with related congeners at length but she clearly opted not to name the taxon due to a lack of additional material. In her caption for her second figure, she wrote “Fig. 2. *Pleistacantha* sp.1, cl.68mm” (Kazmi 1997: 84) while in her table of species discussed, she listed the taxon as “*Pleistacantha* sp.1” (Kazmi 1997: 86). However, in her caption for the first figure of this species, she wrote “Fig. 1. *Pleistacantha adenicus* n. sp., holotype, cl.68 mm” (Kazmi 1997: 83). In the context of her comments, “*Pleistacantha adenicus*” cannot be regarded as an available name. The use of this new name is clearly an accident as her published intentions in the paper are clear. She probably originally wanted to name the new species but decided against this later on before publication, but forgot to remove the name from the first caption. Under the terms of reference for Article 15.1 which states that names regarded as conditionally published after 1960 are not available (ICZN 1999), “*Pleistacantha adenicus* Kazmi, 1997” must therefore be regarded as a nomen nudum.

*Pleistacantha kannu* is probably the same species as Kazmi’s “*P. adenicus*”. Both taxa share the character of the highly inflated branchial regions with the inner margins almost meeting along the midline of the carapace (Kazmi 1997: fig. 1), and their rostral, epistomal, and third maxilliped features (Kazmi 1997: fig. 2A–C) also agree. However, in lieu of examining specimens, and given that her specimen was from the Arabian Sea (the present material is all from southern India), we cannot be certain.

The prominently swollen branchial regions of *Pleistacantha kannu* are noteworthy, and may suggest that they also live in low oxygen deep-sea habitats, an area known as the “oxygen minimum zone” in the Indian Ocean (see Creasey et al. 1997). This is the habitat apparently favoured by the inachid *Encephaloides armstrongi* Wood-Mason, in Wood-Mason & Alcock, 1891, which has even more disproportionately swollen branchial regions, presumably to aid in respiration in such zones (see also Kazmi & Moazzam 2014; Dash et al. 2017). This may also be true of a recently described deep-water homolid, *Moloha tumida* Ng & Kumar, 2015, described also from the western Indian Ocean (Ng and Kumar 2015).

## Supplementary Material

XML Treatment for Pleistacantha

XML Treatment for Pleistacantha kannu
